# Supplementary material for: Altered behaviour and immune response in mice with NHLRC2 p.Asp148Tyr variant
Source: Brain Behav Immun Health. 2025 May 22;46:101020. doi: 10.1016/j.bbih.2025.101020 (PMC12159220; doi:10.1016/j.bbih.2025.101020)
Supplement: Multimedia component 1 [file mmc1.pdf]

Altered behaviour and immune response in mice with NHLRC2 p.Asp148Tyr variant

Authors

Anniina E. Hiltunen, Salla M. Kangas, Aishwarya Gondane, Henna Koivisto, Kari Salokas, Anne Heikkinen, Miia H. Salo, Tapio Röning, Antti Tallgren, Virpi Glumoff, Maria C. Denis, Niki Karagianni, Johanna Myllyharju, Markku Varjosalo, Heikki Tanila, Harri M. Itkonen, Mika Rämet, Johanna Uusimaa, Reetta Hinttala

Supplementary Tables

Supplementary table 1. qPCR primers.

Primer	Sequence (5’-3’)
mRela_F	GGCGGCACGTTTTACTCTTT
mRela_R	CCGTCTCCAGGAGGTTAATGC
Jun_F	GCACATCACCACTACACCGA
Jun_R	GGGAAGCGTGTTCTGGCTAT
mHprt1_F	CTTCCTCCTCAGACCGCTTT
mHprt1_R	CATCATCGCTAATCACGACGC
RPLP0_F	TTGGCCAATAAGGTGCCAGC
RPLP0_R	GGAGGTCTTCTCGGGTCCTA
RPL13A_F	GAGGTCGGGTGGAAGTACCA
RPL13A_R	TGCATCTTGGCCTTTTCCTT
mPramel7_F	GAAAGAGTTGCTCAGGTGTCTG
mPramel7_R	TAAGCTGACTGTAGTTCCAGCG
mSlc27a2_F	CCTCCTGATGATCGACCGTG
mSlc27a2_R	GTTCTCTCCTTTCCACCGGA
mGrb10_F	ACGGAATGCTCCTGTACCAA
mGrb10_R	CAGAAACACTGCGCATAGGTG
mNckap1l_F	TGGACCTCACACAAAGGCTG
mNckap1l_R	ATGAGGCTTAGAAGCTGGGC
Actb_F	GCTGTATTCCCCTCCATCGTG
Actb_R	CACGGTTGGCCTTAGGGTTCAG
Gapdh_F	CCCCAATGTGTCCGTCGTG
Gapdh_R	GCCTGCTTCACCACCTTCT

Supplementary table 2. U-plex analytes and calibrators.

Analyte	Calibrator	Calibrator Cat. Nr.	Calibrator lot
IFN-α	IFN-a	C02W1-2	A00U0216
IFN-β<br>IL-1β<br>IL-2<br>IL-5<br>TNFα<br>GM-CSF	5	C0065-2	A00U0217
IL-17A<br>IL-23	7	C0073-2	A00U0191
IL-15<br>IL-27p28/IL30	8	C00U0192	A00U0192
IP-10<br>MIP-1α	12	C0092-2	A00U0212
IFN-β<br>MDC	16	C0295-2	A00U0187
TARC	17	C0296-2	A00U0223

Supplementary table 3. Pathology scoring for the myelin oligodendrocyte glycoprotein induced experimental autoimmune encephalomyelitis (MOG-EAE) experiment.

Grade	Clinical sign
0	No clinical signs
1	Paralyzed tail
2	Paraparesis (paresis of one or two hind limbs)
3	Complete paralysis of one hind limb
4	Paraplegia (complete paralysis of two hind limbs) and weakness in forelimbs
5	Dead or moribund animal

Supplementary table 4. BD antibodies used in flow cytometric analysis of mouse splenocytes.

Antigen	Cat No	Fluorochrome
CD62L	562910	BV421
B220	563708	BV605
CD120b	550086	PE
CD152/CTLA-4	564332	PE-CF594
CD44	560570	PerCP-Cy5.5
CD3	560591	PE-Cy7
FoxP3	563486	AF647
CD8	557959	Alexa700
CD4	560246	APC-Cy™7
TNF	566287	BV421
IL-17A	564169	BV605
CD25	564458	BB515
IL-4	554389	PE
IL-2	562483	PE-CF594
IFNγ	557735	AF647
Fixable viability	564406	BV510

Supplementary table 5. Other reagents for splenocyte analysis.

Reagent	Cat No	Manufacturer
eBioscience Cell stimulation cocktail (500x)	00-4970	Invitrogen, Thermofisher
eBioscience Cell Stimulation Coctail (plus protein transport inhibitors) (500X)	00-4975	Invitrogen, Thermofisher
eBioscience Protein Transport Inhibitor Coctail (500X)	00-4980	Invitrogen, Thermofisher

Supplementary Figures

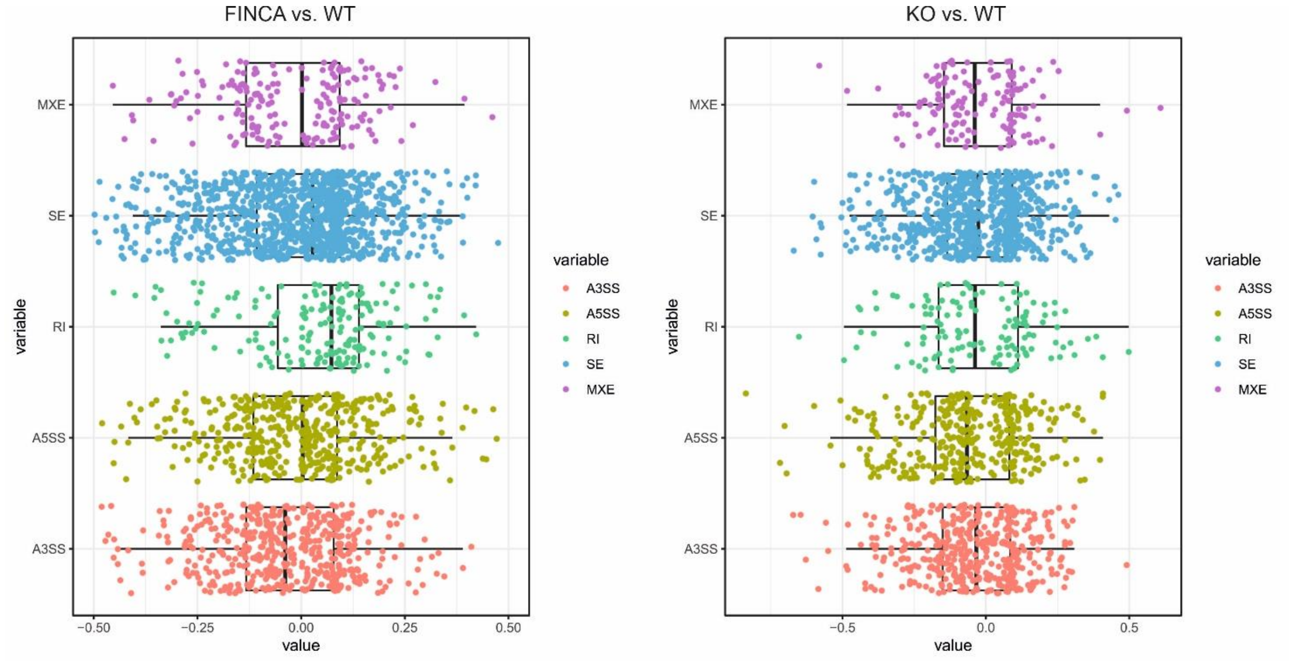

Supplementary Figure 1*. Box blot of significant alternative splicing events (p-value < 0.05) of FINCA variant and KO mES cells compared to WT cells. MXE, mutually exclusive exons; SE, skipped exon; RI, retained intron; A5SS, alternative 5' splice site; A3SS, alternative 5' slice site.*

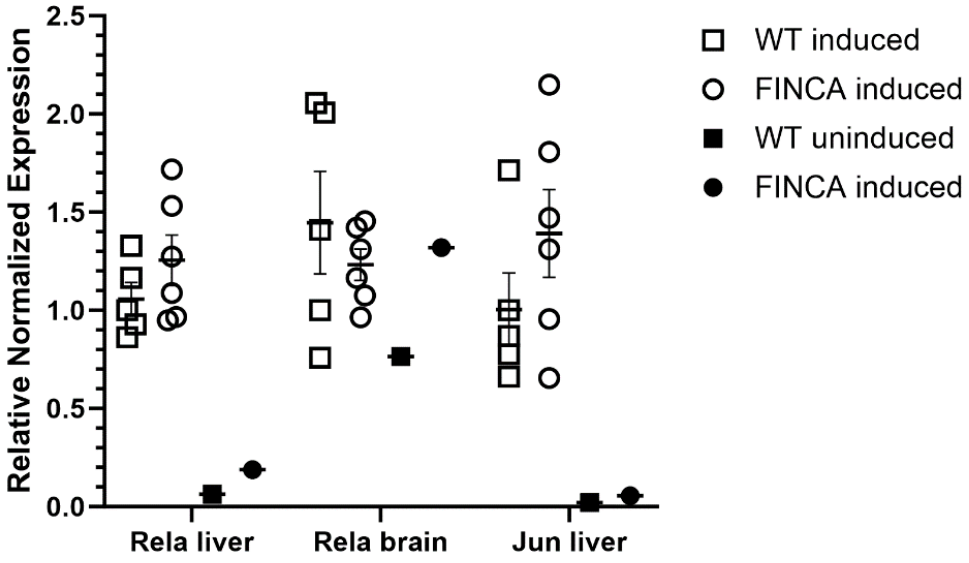

Supplementary Figure 2. *mRNA expression of v-rel reticuloendotheliosis viral oncogene homolog A (avian) (Rela) in brain and liver, and jun proto-oncogene (Jun) expression in liver 6h post LPS injection in 8-week-old FINCA (N = 6) and WT (N = 5) female mice. Uninduced controls were uninjected FINCA (N = 1) and WT (N = 1) mice. Hprt1 and Rplp0 were used as reference genes for liver samples and Hprt1 and Rpl13A were used for brain samples. Statistical analyses were conducted using paired Student's t test, * p < .05, **p < 0.01. Scatter plots show the individual data points, group means, and standard error of means (SEM). WT, wildtype.*

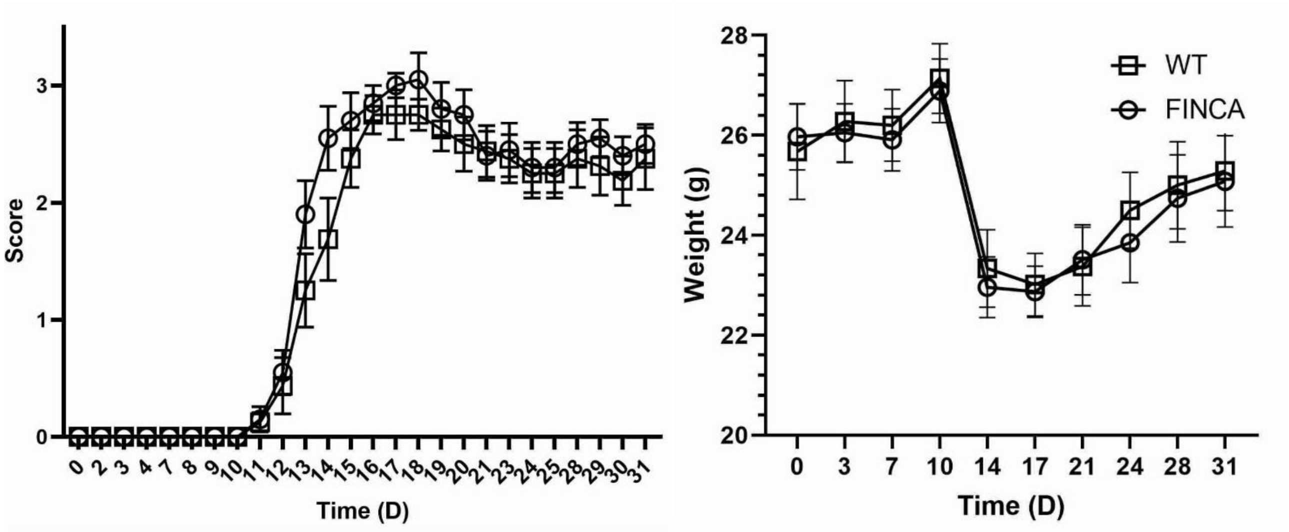

Supplementary Figure 3. *No difference was detected in clinical score or weight during the MOG-EAE experiment between the FINCA (N=10) and WT (N = 8) mice. D, day.*

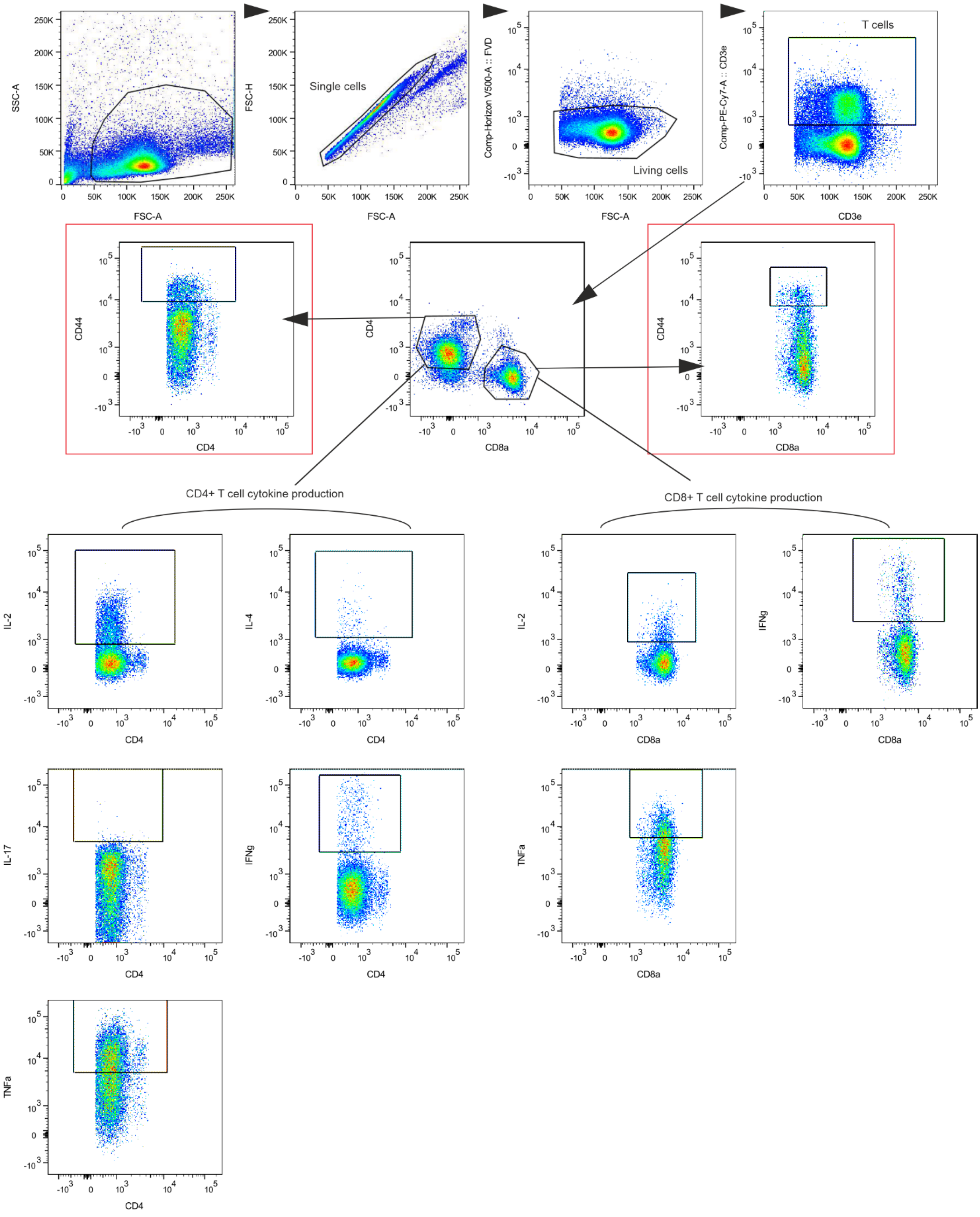

Supplementary Figure 4. *Gating strategy used in flowcytometry to determine cytokine production. The image is a representative image of splenocytes from one WT sample. Red boxes show the cells used for further analysis of CD44 positive CD4+ and CD8+ T-cells by gating, as shown in*

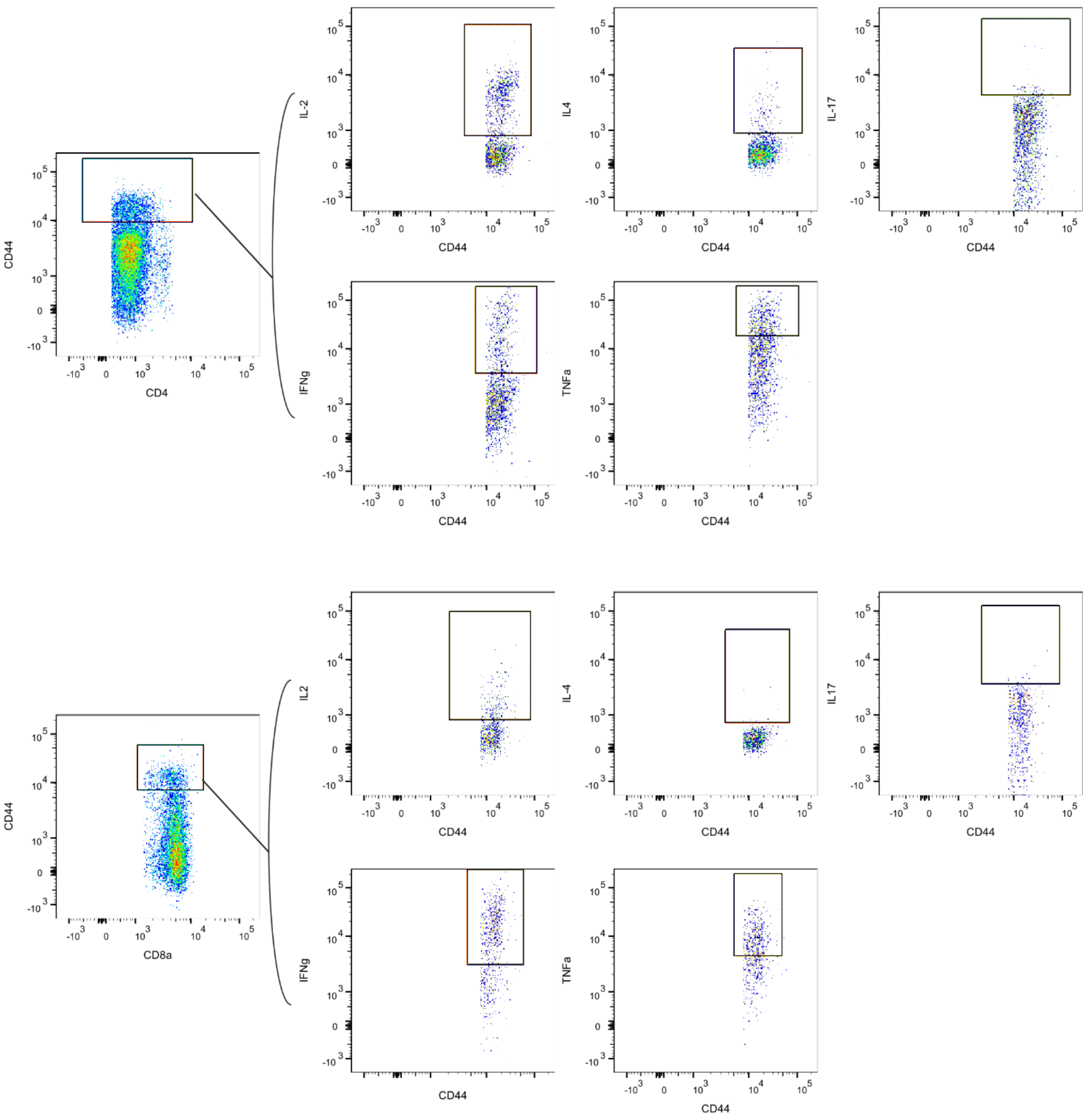

Supplementary figure 5. *Gating used in flowcytometry analysis of cytokine production of CD44 positive CD4+ and CD8+ T-cells. The data shown are from one WT spleen.*

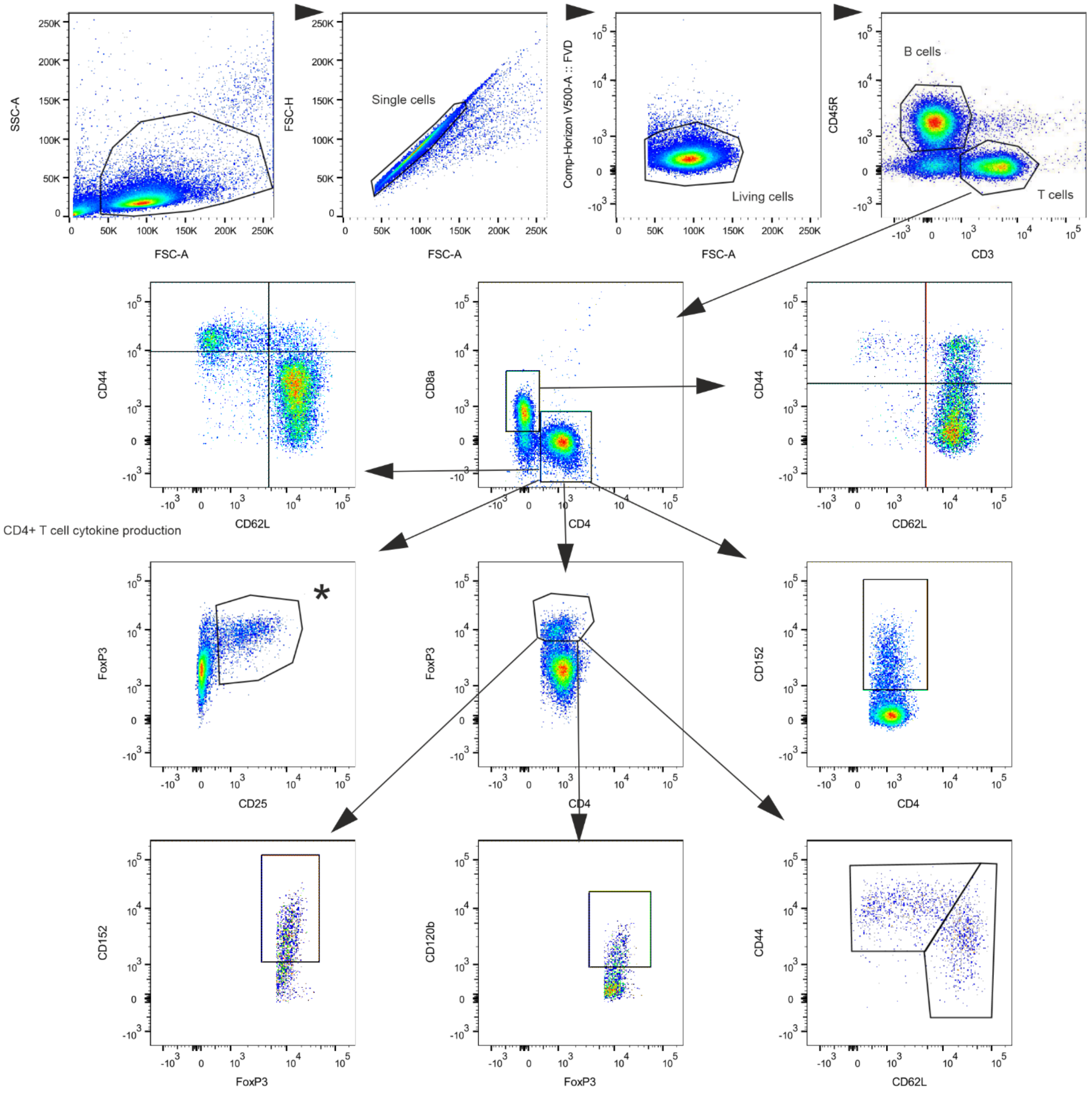

Supplementary figure 6. *Gating used in flowcytometry analysis of different T-cell populations. The data shown are from one WT spleen.*
