# Supplementary material for: Altered behaviour and immune response in mice with NHLRC2 p.Asp148Tyr variant
Source: Brain Behav Immun Health. 2025 May 22;46:101020. doi: 10.1016/j.bbih.2025.101020 (PMC12159220; doi:10.1016/j.bbih.2025.101020)
Supplement: Multimedia component 4 [file mmc4.docx]

**Mmc1. Supplementary information**

Supplementary tables: Table S1. qPCR primers. Table S2. U-plex analytes and calibrators. Table S3. Pathology scoring for the myelin oligodendrocyte glycoprotein induced experimental autoimmune encephalomyelitis (MOG-EAE) experiment. Table S4. BD antibodies used in flow cytometric analysis of mouse splenocytes. Supplementary figures: Figure S1. Box blot of significant alternative splicing events. Figure S2. mRNA expression of v-rel reticuloendotheliosis viral oncogene homolog A (avian) (Rela) in brain and liver, and jun proto-oncogene (Jun) expression in liver 6h post LPS injection in 8-week-old FINCA (N = 6) and WT (N = 5) female mice. Figure S3. No difference was detected in clinical score or weight during the MOG-EAE experiment between the FINCA (N=10) and WT (N = 8) mice. Figure S4. Gating strategy used in flowcytometry to determine cytokine production. Figure S5. Gating used in flowcytometry analysis of cytokine production of CD44 positive CD4+ and CD8+ T-cells. Figure S6. Gating used in flowcytometry analysis of different T-cell populations.

**Mmc2. Supplementary data 1**

Differentially expressed genes between FINCA and WT mESCs, and between Nhlrc2 KO and WT mESCs, gProfiler version used for pathway and gene enrichment analysis. Pathway and gene enrichment analysis results of FINCA DEGs, DEGs with increased expression due to FINCA variant, DEGs with decreased expression due to FINCA variant, and of Nhlrc2 KO DEGs

**Mmc3. Supplementary data 2**

List of proteins with reduced and increased interaction with the NHLRC2 p.Asp148Tyr variant compared to wild type NHLRC2 according to Proximity-Dependent Biotin Identification analysis in HEK cells, list of common genes of which transcription was affected by the p.Asp148Tyr variant in mESCs and interaction was affected in BioID2 of HEK 293 cells, gProfiler version used pathway and gene enrichment analysis, Pathway and gene enrichment analysis of hits identified in BioID and of hits with decreased interaction in BioID and increased expression in RNAseq.
